# H_2_S-driven chemotherapy and mild photothermal therapy induced mitochondrial reprogramming to promote cuproptosis

**DOI:** 10.1186/s12951-024-02480-x

**Published:** 2024-04-24

**Authors:** Lihong Qiao, Yijing Ou, Lin Li, Shuzhen Wu, Yanxian Guo, Mu Liu, Dongsheng Yu, Qinghua Chen, Jianmin Yuan, Chuanqi Wei, Chiyi Ou, Haowen Li, Du Cheng, Zhiqiang Yu, Zhongjun Li

**Affiliations:** 1 Department of Obstetrics and Gynecology, The Tenth Affiliated Hospital of Southern Medical University (Dongguan People’s Hospital), Dongguan, Guangdong 523058 People’s Republic of China; 2 Dongguan Key Laboratory of Major Diseases in Obstetrics and Gynecology, The Tenth Affiliated Hospital of Southern Medical University (Dongguan People’s Hospital), Dongguan, Guangdong 523058 People’s Republic of China; 3 Department of Laboratory Medicine Dongguan Institute of Clinical Cancer Research, The Tenth Affiliated Hospital of Southern Medical University (Dongguan People’s Hospital), Dongguan, Guangdong, 523058 People’s Republic of China; 4 Key Laboratory for Polymeric Composite & Functional Materials of Ministry of Education, School of Materials Science and Engineering, Sun Yat-sen University, Guangzhou, Guangdong, 510275 People’s Republic of China

**Keywords:** Cuproptosis, H_2_S-responsive, Mitochondrial reprogramming, Mild photothermal therapy, Chemodynamic therapy

## Abstract

The elevated level of hydrogen sulfide (H_2_S) in colon cancer hinders complete cure with a single therapy. However, excessive H_2_S also offers a treatment target. A multifunctional cascade bioreactor based on the H_2_S-responsive mesoporous Cu_2_Cl(OH)_3_-loaded hypoxic prodrug tirapazamine (TPZ), in which the outer layer was coated with hyaluronic acid (HA) to form TPZ@Cu_2_Cl(OH)_3_-HA (TCuH) nanoparticles (NPs), demonstrated a synergistic antitumor effect through combining the H_2_S-driven cuproptosis and mild photothermal therapy. The HA coating endowed the NPs with targeting delivery to enhance drug accumulation in the tumor tissue. The presence of both the high level of H_2_S and the near-infrared II (NIR II) irradiation achieved the in situ generation of photothermic agent copper sulfide (Cu_9_S_8_) from the TCuH, followed with the release of TPZ. The depletion of H_2_S stimulated consumption of oxygen, resulting in hypoxic state and mitochondrial reprogramming. The hypoxic state activated prodrug TPZ to activated TPZ (TPZ-ed) for chemotherapy in turn. Furthermore, the exacerbated hypoxia inhibited the synthesis of adenosine triphosphate, decreasing expression of heat shock proteins and subsequently improving the photothermal therapy. The enriched Cu^2+^ induced not only cuproptosis by promoting lipoacylated dihydrolipoamide S-acetyltransferase (DLAT) heteromerization but also performed chemodynamic therapy though catalyzing H_2_O_2_ to produce highly toxic hydroxyl radicals ·OH. Therefore, the nanoparticles TCuH offer a versatile platform to exert copper-related synergistic antitumor therapy.

**Supplementary Information:**

The online version contains supplementary material available at 10.1186/s12951-024-02480-x.

## Introduction

Colon cancer has the third highest prevalence among common malignancies, as well as the second highest mortality rate. Conventional treatment methods (e.g., surgical resection and chemotherapy) are widely used in the management of colon cance. However, chemotherapy alone has considerable toxic side effects and can cause damage to normal tissues and cells. It also has an increased risk of inducing drug resistance, eventually leading to tumor recurrence and metastasis [3–5]. Almost 90% of cancer related deaths are caused by metastasis, rather than growth of the primary tumor. Additionally, the colon cancer microenvironment contains complex physiological systems, such as increased levels of the endogenous hydrogen sulfide (H_2_S, ∼3.4 mmol), hydrogen peroxide (H_2_O_2_, ∼10 mmol) and glutathione (GSH, ∼10 mmol), which may limit therapeutic efficacy [6–9]. There are two main sources of endogenous H_2_S. The first source comprises increased expression of the cystathionine-β-synthase enzyme, which catalyzes H_2_S production from sulfur-containing compounds in colon cancer [10, 11]. The second source comprises sulfur-reducing microorganisms, such as *Fusobacterium nucleatum*, which can catalyze the decomposition of sulfur-containing amino acids into H_2_S in the tumor microenvironment, leading to excess H_2_S [12]. Recent studies have shown that long-term exposure to H_2_S does not impair viability in normal cells. Consequently, endogenous H_2_S-responsive nanoparticles (NPs) are becoming attractive targets for the development of stimulus-responsive therapeutic models [13–16].

In recent years, when treating tumors in the complex microenvironment of colon cancer, the use of a single treatment method has led to unsatisfactory patient outcomes [17–21]. Therefore, considerable effort has been invested in combining multiple individual treatment components to produce optimized treatment methods. The increased levels of endogenous H_2_S, as well as high-affinity interactions with Cu^2+^, can be utilized to produce copper sulfide (Cu_9_S_8_) in situ via near-infrared II as photothermal therapy (PTT) for colon cancer [22, 23]. However, because protein heat tolerance is a natural physiological response, the effects of PTT are limited by the upregulation of heat shock proteins (HSPs) in the tumor microenvironment. Thus, mild PTT is not widely useful in cancer treatment [24]. Conveniently, the consumption of large amounts of H_2_S promotes mitochondrial reprogramming, and the resulting decrease in oxygen helps to activate anoxic drugs, generating active drugs [25–27] and providing the required conditions for effective hypoxic prodrugs (such as tirapazamine [TPZ]). Importantly, HSPs are key proteins in the induction of cellular thermotolerance; these chaperone proteins function in an ATP-binding and hydrolysis-dependent manner. H_2_S-induced intracellular mitochondrial reprogramming leading to ATP depletion is expected to block HSP production, thereby reversing PTT resistance in tumors. These changes help to alleviate damage to normal tissues and cells around the tumor, while inducing inflammation [28].

Cuproptosis is a type of non-apoptotic cell death regulated by copper dependence, which differs from previously identified death patterns [29–32]. Copper is also a trace metal necessary for the proper function of metabolic enzymes, but excessive amounts of copper can lead to increased levels of free radicals in cells that rely on mitochondrial respiration, followed by apoptosis [33]. Copper-dependent death is caused by the direct binding of copper to lipidated components of the tricarboxylic acid (TCA) cycle. This binding leads to the aggregation of fatty proteins and subsequent loss of iron-sulfur cluster proteins, followed by proteotoxic stress and cell death. The enrichment of copper ions in the tumor also provides the conditions necessary for chemodynamic therapy (CDT). Thus, an H_2_S-responsive Cu^2+^ multifunctional cascade bioreactor may have substantial efficacy for induced mitochondrial reprogramming-based synergistic anticancer treatment [34–38].

**Scheme 1:**
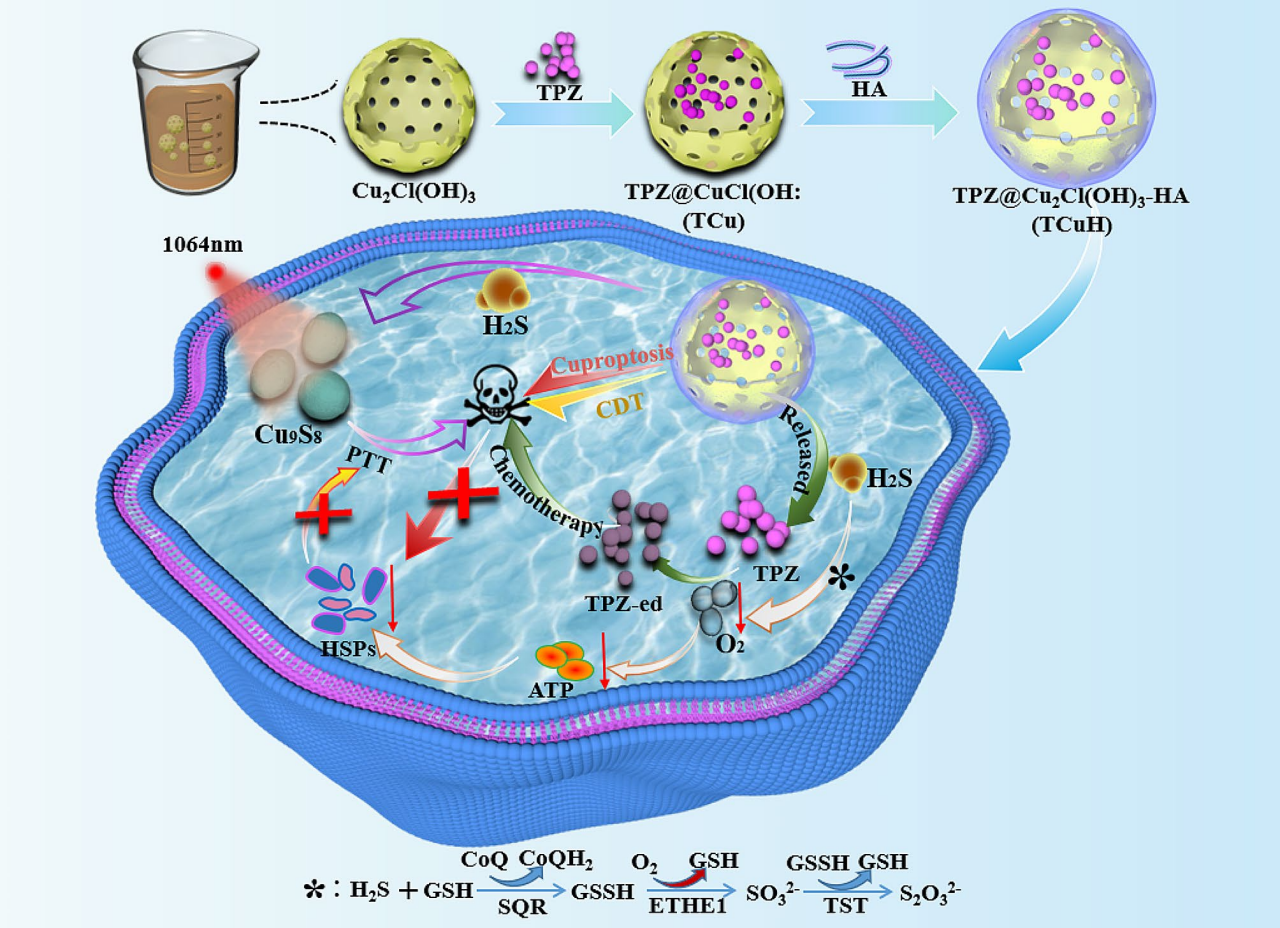
Schematic illustration of the H_2_S-driven chemotherapy and mild photothermal therapy induced mitochondrial reprogramming to promote cuproptosis

Here, we developed an H_2_S-driven chemotherapy and mild photothermal therapy induced mitochondrial reprogramming to promote cuproptosis. In this approach, the hypoxic prodrug TPZ was loaded into mesoporous Cu_2_Cl(OH)_3_ to form a TCuH nanomaterial with targeting function and low toxicity; it was then coated with hyaluronic acid (HA) to achieve high hydrophilicity while maintaining the targeting function. TCuH NPs were injected through the caudal vein and concentrated in colon cancer through their enhanced permeability, retention effects, and active targeting. TCuH reacted with excess H_2_S and near-infrared II (NIR II) in the tumor microenvironment to form copper sulfide (Cu_9_S_8_) and release the hypoxic prodrug TPZ (Scheme 1). When H_2_S content was sufficiently depleted, the induction of mitochondrial reprogramming stimulated oxygen consumption by colonic epithelial cells. As in the previous works noted above, H_2_S oxidation in mitochondria altered cellular bioenergetics, inducing reductive transition that involved NAD/NADH redox pairs. Subsequently, electron receptor deficiency led to deficiencies in uridine and L-aspartic acid and the enhancement of L-glutamine-dependent reductive carboxylation, thereby exacerbating hypoxia in the tumor microenvironment. Activation of the hypoxic prodrug TPZ yielded activated TPZ (TPZ-ed) for chemotherapeutic treatment of colon cancer. Further exacerbation of hypoxia inhibited the synthesis of ATP, leading to decreased expression of HSPs and improving the effectiveness of mild PTT treatment. Additionally, copper ions were enriched in colon cancer, and Cu^2+^ bound to lipoacylated dihydrolipoamide S-acetyltransferase (DLAT), inducing DLAT heteromerization. The increase in insoluble DLAT led to cytotoxicity and cell death, followed by cuproptosis. Simultaneously, Cu^2+^ generated highly catalytic Cu^+^ under conditions of increased GSH expression, which then catalyzed H_2_O_2_ to produce highly toxic hydroxyl radicals (·OH) during CDT, inducing apoptosis.

## Experimental section

### Synthesis of mesoporous Cu_2_Cl(OH)_3_

Briefly, CuCl_2_·2H_2_O (0.17 g, 1.0 mmol) was placed in a beaker (500 mL) containing 200 mL ethanol, and 1 M NaOH (1.0 mL) was added. Then, 1 mL H_2_O_2_ was slowly added to the beaker; the beaker was covered by cling film with multiple pores. Thereafter, the beaker was placed in a vacuum drying chamber at 30 °C for 72 h. After the reaction was completed, crude product was obtained by centrifugation at 10,000 rpm for 10 min; it was washed three times with distilled water to obtain mesoporous Cu_2_Cl(OH)_3_ as the final product.

### Synthesis of TPZ@Cu_2_Cl(OH)_3_ (hereinafter abbreviated as TCu)

TPZ (1 mg) and mesoporous Cu_2_Cl(OH)_3_ (5 mg) were added into glass bottles with 10 mL of distilled water. The reaction proceeded at room temperature for 4 h. Then, TCu was obtained by centrifugation at 10,000 rpm for 10 min.

### Synthesis of TPZ@Cu_2_Cl(OH)_3_-HA (hereinafter abbreviated as TCuH)

TCu (2 mg) and HA (4 mg/mL, 1 mL) were also prepared by repeating the above steps and centrifuging to obtain the final product: TCuH.

### Synthesis of ICG@Cu_2_Cl(OH)_3−_HA or Cu_2_Cl(OH)_3_@HA

Refer to the TCuH section for synthesis steps. Named ICuH or CuH.

### Stimulus response release

In vitro, NaHS (instead of H_2_S) reacted with mesoporous Cu_2_Cl(OH)_3_ to produce Cu_9_S_8_ and release TPZ. TCuH (2 mg/mL, 1 mL) was placed in a small glass vial with a solution of NaHS (3.7 mmol). The consumption of NaHS and release of TPZ were recorded by UV-Vis spectroscopy and TEM.

### Photothermal capability of Cu_9_S_8_

TCuH (2 mg/mL, 1 mL) and NaHS (3 mmol) were reacted to produce the photothermal agent Cu_9_S_8_. Cu_9_S_8_ (0 ?∼ 100 µg/mL) was excited using a 1064 nm laser, and the photothermal conversion efficiency of Cu_9_S_8_ was calculated. The photothermal properties of Cu_9_S_8_ were examined at various laser powers (0.25, 0.5, and 1.0 w).

### Hydroxyl radical (·OH) detection

During in vitro simulation of ·OH production, a pseudo-Fenton reaction rapidly oxidized methylene blue (MB) via •OH. First, TCuH (2 mg/mL, 1 mL) and GSH (10 mmol) were reacted for 5 min; the solution was collected and placed into a centrifuge tube with H_2_O_2_ (10 mmol). The absorbance at 660 nm was determined using UV-Vis spectroscopy from 0 to 30 min.

### MTT (3-(4,5-dmethylthiazol-2-yl)-2,5-diphenyltetrazolium bromide) assay

CT26 mouse colon carcinoma cells (purchased from the Institute of Biochemistry and Cell Biology, Chinese Academy of Sciences, Shanghai, China) were incubated in RPMI 1640 medium with 10% fetal bovine serum and 1% penicillin–streptomycin (10,000 U/mL); they were cultured in a humidified atmosphere with 5% CO_2_ at 37 °C. For analysis, CT26 cells were seeded in 96-well plates at a density of 10^5^ cells/well and incubated in RPMI 1640 medium for 24 h. The medium was then replaced with PBS, TPZ, CuH, TCuH, and TCuH + 1064 nm at concentrations of 0 to 200 µg/mL. Then, MTT (5 mg/mL, 20 µL) solution was added to each plate and cells were incubated at 37 °C for 4 h.

### Intracellular detection of ROS in vitro

2′,7′-dichlorofluorescin diacetate (DCFH-DA) was used to evaluate ROS production via confocal laser scanning microscope (CLSM) quantification. CT26 cells grown in 6-well plates were treated with PBS, TPZ, CuH, TCuH, and TCuH + 1064 nm laser. After incubation for 4 h, CT26 cells were stained with DCFH-DA to determine ROS production in vitro by CLSM (Leica SP8, Germany).

### Intracellular GSH depletion

CT26 cells grown in 6-well plates were treated with PBS, TPZ, CuH, TCu, and TCuH + 1064 nm laser. After incubation for 4 h, CT26 cells were assayed used a proprietary non-fluorescent dye (Abcam, USA) that can strongly fluoresce upon reaction with GSH; the results were recorded using a microplate reader (Ex/Em = 490/520 nm).

### Biodistribution of TCuH

TCuH biodistribution was examined in BALB/c nude mice (female, 4 weeks old, body weight 15 ?∼ 20 g). ICG (instead of TPZ) was used to produce ICuH, which was intravenously injected into the mice at various times (0, 2, 4, 8, 12, 24 h). The mice were sacrificed after the experiment; tumors and the heart, liver, spleen, lungs, and kidneys were harvested for imaging.

### Western blotting analysis

HSP70, HSP90, FDX1, hypoxia, HIF-1a, and GAPDH in CT26 cells were analyzed by western blotting analysis after treatment with PBS, TPZ, CuH, TCu, and TCuH + 1064 nm for 24 h. Cell lysates were collected and analyzed by 12% denaturing polyacrylamide gel electrophoresis.

### In vivo treatment effect evaluation

Hela cells were implanted into five weeks old female BALB/c nude subcutaneous with body weight for 15 ?∼ 20 g. In briefly, PBS (100 µL) containing 5.0 × 10^6^ Hale cells was injected subcutaneously into the right forelimb of mice. After 5 d of tumor transplantation, the mice were randomly divided into five groups with 5 mice in each group receiving treatments of PBS, TPZ, TCu, TCuH, and TCuH + 1064 nm laser, respectively. Treatments were administered through the tail vein, once every other day for five total treatments; the total dose was 5 mg/kg. Meanwhile, body weights and tumor volume of mice were recorded every 2 d. After 14 days of treatment, the mice were killed and the tumors as well as major organs and tissues were dissected for subsequent efficacy and safety studies.

## Results and discussion

### Preparation and characterization of TCuH nanoformulations

**Fig. 1:**
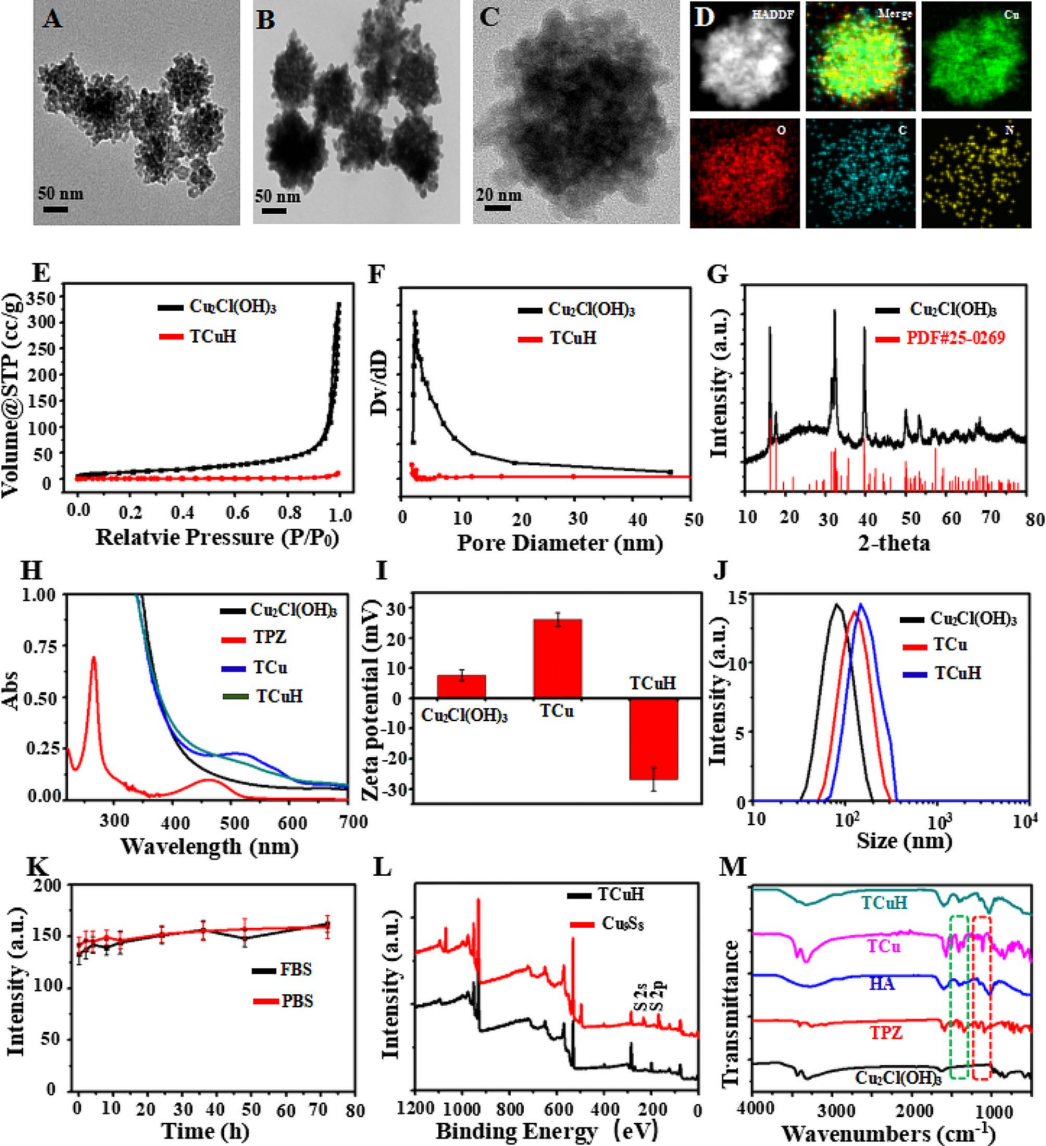
Physical and chemical characterization of Cu_2_Cl(OH)_3_ and TCuH NPs. TEM images of Cu_2_Cl(OH)_3_ NPs (**A**) and TCuH NPs (**B** and **C**). (**D**) Scanning TEM image and corresponding elemental mappings of Cu, C, and N in TCuH NPs. (**E**) N_2_ adsorption/desorption isotherm of Cu_2_Cl(OH)_3_ NPs. (**F**) Pore size distribution of modified Cu_2_Cl(OH)_3_ NPs in N_2_ adsorption/desorption isotherm. (**G**) X-ray diffraction pattern of Cu_2_Cl(OH)_3_ NPs. (**H**) UV-vis spectroscopy of Cu_2_Cl(OH)_3_, TPZ, TCu and TCuH NPs. (**I**) Zeta potentials of Cu_2_Cl(OH)_3_, TCu and TCuH NPs. (**J**) Size distributions of Cu_2_Cl(OH)_3_, TCu and TCuH NPs. (**K**) Test of TCuH NP stability in fetal bovine serum and PBS for 72 h. (**L**) XPS of TCuH NPs before and after treatment with NaHS. (M) Fourier-transform infrared spectra of TPZ, HA, Cu_2_Cl(OH)_3_, TCu and TCuH NPs

Mesoporous Cu_2_Cl(OH)_3_ was synthesized with CuCl_2_·2H_2_O, NaOH, and H_2_O_2_ by a standard gentle method (see Supporting Information for the detailed protocol). After centrifugation, mesoporous Cu_2_Cl(OH)_3_ NPs with uniform particle size were obtained. As shown in Fig. 1A, transmission electron microscopy (TEM) analyses revealed that mesoporous Cu_2_Cl(OH)_3_ had a size of 76 nm. Next, the hypoxic prodrug TPZ was sequentially adsorbed into mesoporous Cu_2_Cl(OH)_3_ by mechanical stirring, generating TCu. HA was then wrapped in TPZ@Cu_2_Cl(OH)_3_ (TCu) to yield TPZ@Cu_2_Cl(OH)_3_-HA (TCuH) NPs. Figure 1B and C are TEM images of TCuH NPs, which had a uniform size of 132 nm. Elemental mapping (Fig. 1D) via scanning TEM showed that Cu, O, C, and N were uniformly distributed in the TCuH NPs. Energy-dispersive spectroscopy (EDS) displayed a similar elemental distribution (Figure SI 1). Brunauer–Emmett–Teller images (Fig. 1E and F) revealed the porous structure of mesoporous Cu_2_Cl(OH)_3_ via N_2_ adsorption isotherm analysis. Adsorption hysteresis confirmed the porous structure of mesoporous Cu_2_Cl(OH)_3_. The surface area, pore volume and pore size were 63.56 m^2^/g, 0.02 cm^3^/g and 2.91 nm, respectively. However, Both the surface area and pore volume were much lower, i ncluding the pore size. Because Cu_2_Cl(OH)_3_ was coated by HA, almost no pore size of mesopore Cu_2_Cl(OH)_3_ was measured, indicating that HA was coated on pore size. The crystalline nature was confirmed by X-ray diffraction (Fig. 1G), which showed Cu_2_Cl(OH)_3_ (PDF#25–0269). The TPZ characteristic absorbance peak at 518 nm in the UV-Vis spectrum of TCu (Fig. 1H) was weakly detectable because of its high HA content. The TPZ loading content was 8.7%, according to UV-Vis (Figure SI 2). The zeta potentials of + 7.6 mV (Cu_2_Cl(OH)_3_), + 26.1 mV (TCu), and − 26.8 mV (TCuH) confirmed successful loading of TPZ and HA onto Cu_2_Cl(OH)_3_ (Fig. 1I). According to dynamic light scattering, the hydrodynamic diameters of Cu_2_Cl(OH)_3_, TCu, and TCuH were 88 nm, 103 nm, and 144 nm, respectively (Fig. 1J). Changes in average particle size were negligible after incubation with 10% fetal bovine serum in water and PBS for 72 h. As shown in Fig. 1K, TCuH maintained good water dispersibility and no visible precipitation; thus, it was stable in a solution of 10% fetal bovine serum in water. Additionally, the composition of TCuH NPs was studied by X-ray photoelectron spectroscopy (XPS, black line), which revealed four characteristic peaks at 285.86 eV (C1s), 399.48 eV (N1s), 532.73 eV (O1s), and 934.02 eV (Cu 2p). Importantly, the valence state of Cu 2p is shown in Figure SI 3; the results confirmed that Cu^2+^ had reacted with GSH and been reduced to Cu^+^, which could catalyze H_2_O_2_ generation from ·OH through a Fenton-like reaction during CDT. In the Fourier-transform infrared spectrum of TCuH (Fig. 1M), the presence of the characteristic peaks of TPZ and Cu_2_Cl(OH)_3_ suggested successful binding between TPZ and Cu_2_Cl(OH)_3_. Furthermore, the characteristic peaks of TCuH NPs were similar to HA because of high HA content in the outer layer.

### Sulfidation of TCuH NPs and release of TPZ

**Fig. 2:**
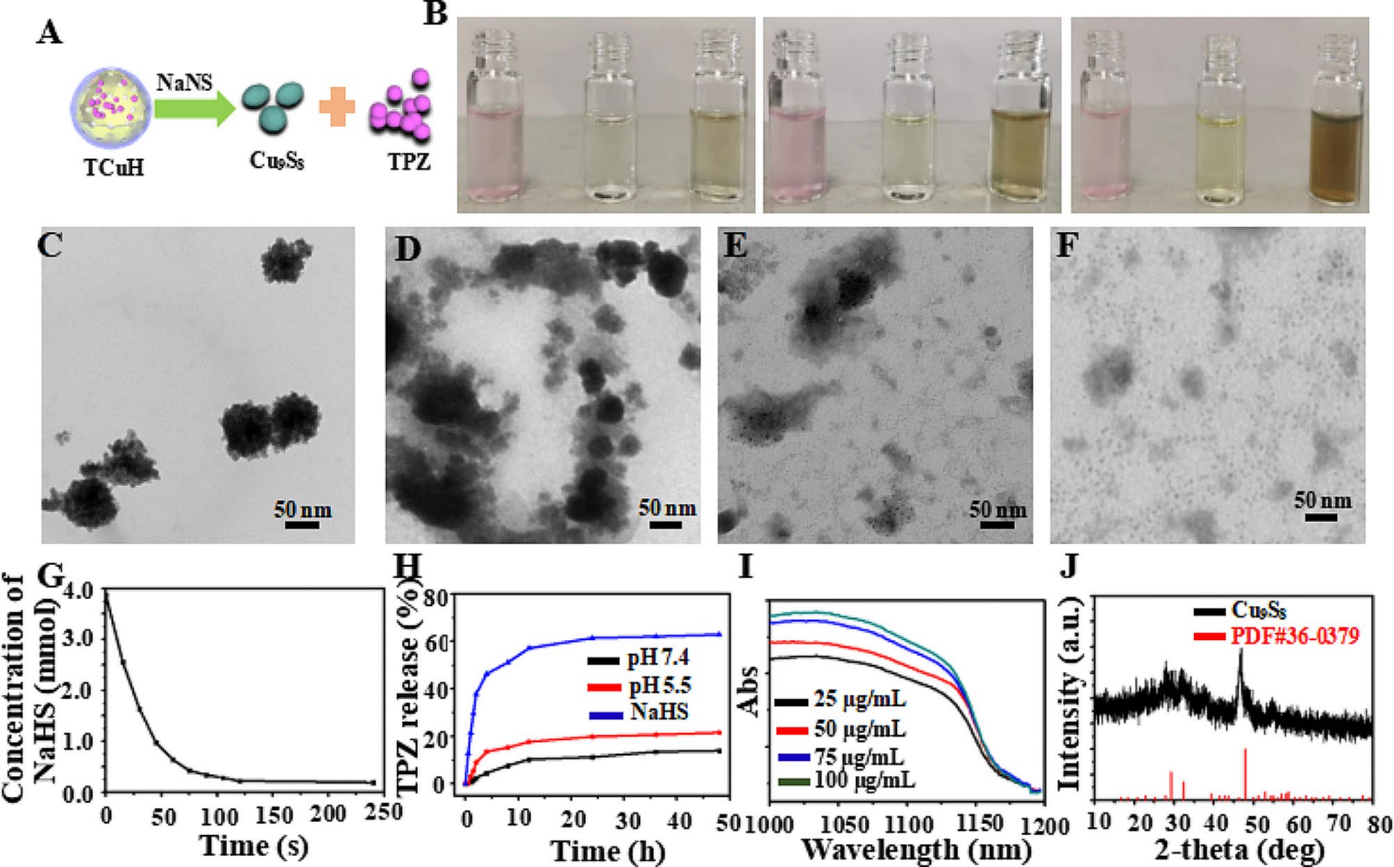
Characterization of TCuH NPs in aqueous solution and performance in the presence of NaHS. (**A**) Schematic diagram of TCuH and NaHS reacting to generate the photothermal agent Cu_9_S_8_ and release the hypoxia prodrug TPZ. (**B**) Photographs of TCuH NPs at various concentrations in the presence of NaHS. (**C**-**F**) TEM images of TCuH NPs reacted with various concentrations of NaHS (0, 0.5, 1, and 3 mmol). (**G**) NaHS consumption over time according to TCuH concentration in vitro. (**H**) TPZ release over time in the presence of pH 7.4, pH 5.5 and NaHS, respectively. (**I**) UV-Vis spectra of TCuH NPs after incubation with various concentrations of NaHS. (**J**) X-ray diffraction patterns of reaction products of TCuH NPs after treatment with NaHS (50 µg/mL) for 30 min

Chemical reactions with TCuH were simulated using sodium hydrosulfide (NaHS) as a H_2_S-rich microenvironment *in vitro.* Figure 2A shows the reaction of TCuH with NaHS to produce Cu_9_S_8_ (PDF#36–0379, Fig. 1G red line) and release the prodrug TPZ. As indicated in Fig. 2B, the solution color rapidly changed from pink to brown when TCuH reacted with various concentrations of NaHS (0, 0.5, 1, and 3 mmol). Additionally, TCuH reacted with NaHS (3 mmol) at various time intervals to achieve TCuH cleavage, as determined by TEM (Fig. 2C-F). The consumption of NaHS was recorded by UV-Vis, as shown in Fig. 2G; NaHS quantities below 20 µmol were rapidly consumed by TCuH. This consumption provided the conditions that initiated the reaction in Scheme 1*, which reduced oxygen levels at the tumor site. In the hypoxic environment, TPZ was activated to produce the chemotherapeutic drug TPZ-ed. TCuH was subsequently cleaved by NaHS (3 mmol) to release TPZ (Fig. 2H). More than 50% of TPZ was released within 10 h. However, the release of TPZ in PBS solutions with pH 5.5 and 7.4 was less than 20%, far less than that in NaHS solutions. Indicating that NaHS reacted more easily with TCuH to release TPZ. Additionally, the absorption intensity in the near-infrared region increased with increasing Cu_9_S_8_ concentration (25, 50, 75 and 100 µg/mL, Fig. 2I), demonstrating that TCuH NPs could react with NaHS. Moreover, the Cu_2_Cl(OH)_3_ NPs and NaHS reacted for 30 min to produce Cu_9_S_8_, which was consistent with PDF#36–0379 (Fig. 2J).

### In Vitro simulation OH production and photothermal effect

**Fig. 3:**
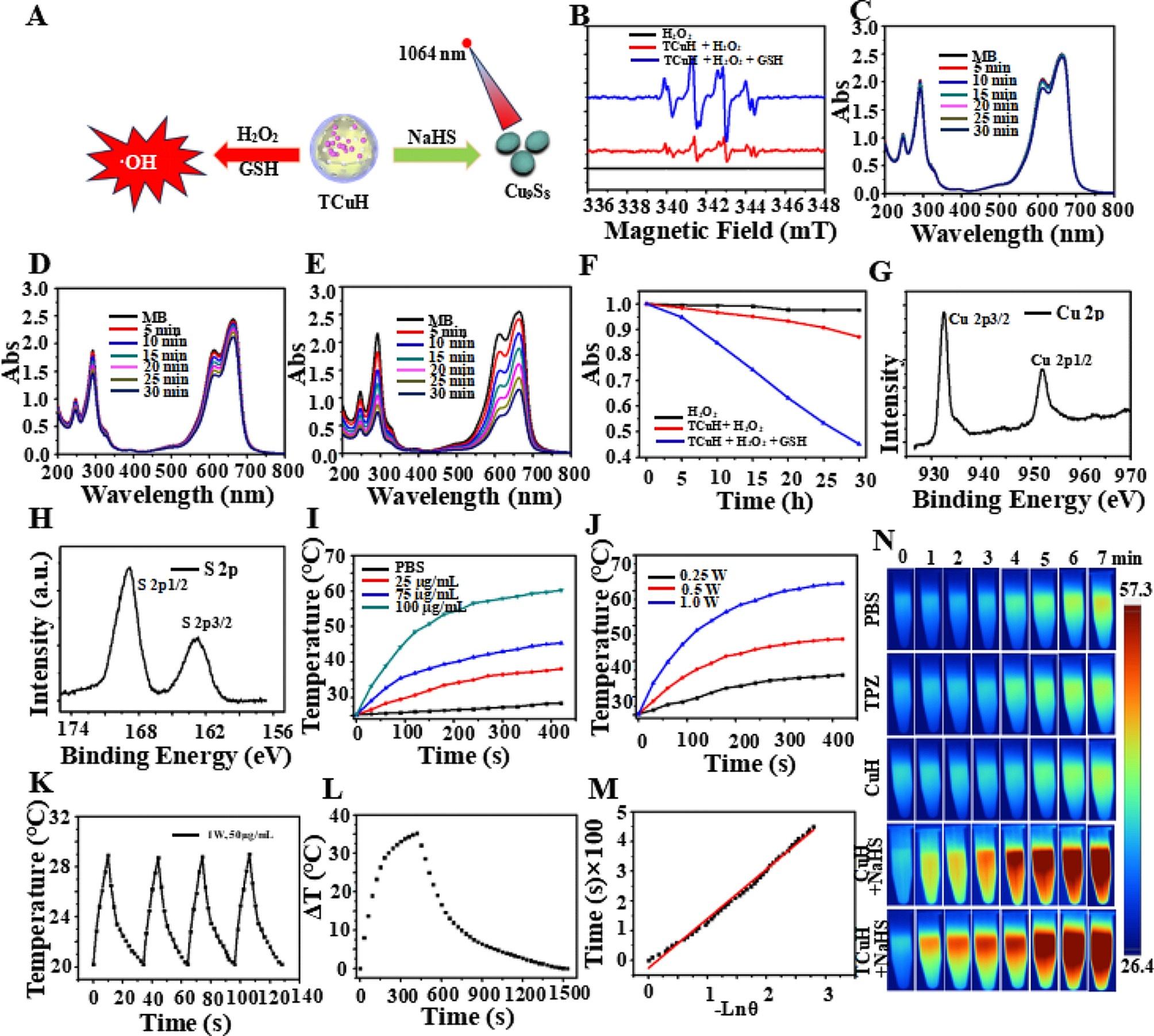
CDT with TCuH NPs in an H_2_O_2_/GSH environment and photothermal performances of TCuH after incubation with various concentrations of NaHS. (**A**) Schematic diagram of TCuH and NaHS reacted to generate photothermal agent Cu_9_S_8_ and TCuH reduction to Cu^+^ by excess GSH, which catalyzed production of •OH from excess H_2_O_2_. (**B**) Electron spin resonance spectra of TCuH NPs (10 µg/m) with < 10 mmol GSH/H_2_O_2_. (**C**) Time-dependent UV-Vis absorption spectra of MB-containing TCuH NPs (10 µg/mL) in solution without GSH/H_2_O_2_. (**D**) Time-dependent UV-Vis absorption spectra of MB-containing TCuH NPs (10 µg/mL) in solution with GSH (10 mM) and without H_2_O_2_. (**E**) Time-dependent UV-Vis absorption spectra of MB-containing TCuH NPs (10 µg/mL) in solution with GSH/H_2_O_2_ (10 mmol). (**F**) Normalized absorbance of the MB solution in the presence of TCuH NPs (10 µg/mL) with/without the addition of H_2_O_2_ and GSH. (**G**) HR-XPS of Cu 2p (Cu_9_S_8_). (**H**) HR-XPS of S 2p (Cu_9_S_8_). (**I**-**M**) Representative temperature evolution curves of aqueous dispersions of TCuH NPs at various concentrations (25, 75, and 100 µg/mL) in the presence of NaHS under 1064 nm laser irradiation at 1.0 W/cm^2^. (**N**) Corresponding thermal images of TCuH NPs (100 µg/mL) in the presence of NaHS (3 mmol) under 1064 nm laser irradiation for various times (0–7 min)

The sulfurization reaction and Fenton-like reaction were simulated in vitro at high levels of GSH (?∼ 10 mmol), H_2_O_2_ (?∼ 10 mmol), and H_2_S (?∼ 3.4 mmol) to produce greater intracellular oxidative stress (Fig. 3A). Cu^2+^ was reduced to Cu^+^ by excess GSH, which catalyzed the production of •OH from excess H_2_O_2_. First, •OH production was confirmed by using 5,5-dimethyl-1-pyrroline-N-oxide (DMPO) as a free radical trapping agent for electron spin resonance spectroscopy analysis. As shown in Fig. 3B, there were obvious characteristic peaks (i.e., 1:2:2:1 four-line signal) of •OH in the electron spin resonance analysis, indicating that Cu^+^ could catalyze the production of •OH from H_2_O_2_. Moreover, methylene blue (MB) was utilized as a monitoring agent to quantitatively analyze the generation of •OH by UV-Vis. Blue MB was oxidized by •OH to a colorless cationic radical, resulting in reduced UV-Vis absorption at 665 nm. Time-dependent UV-Vis absorption spectra of MB-containing TCuH (30 µg/ml) solution were obtained without GSH and H_2_O_2_ (Fig. 3C). There were minimal changes in peak intensity when TCuH (30 µg/mL) was reacted with H_2_O_2_ (10 mM), indicating limited •OH production (Fig. 3D). TCuH was reacted with GSH (10 mM) for 5 min and then with H_2_O_2_ (10 mmol) to measure time-dependent UV-Vis absorption spectra. As shown in Fig. 3E, the absorption intensity of MB considerably decreased, demonstrating substantial •OH production. Figure 3F was obtained from the normalized data. Notably, a large amount of •OH was only generated in the presence of GSH (10 mmol) and H_2_O_2_ (10 mmol). Next, we explored the reaction of TCuH (30 µg/mL) with NaHS (3 mmol) to produce Cu_9_S_8_, along with its photothermal properties. As shown in Fig. 1L, compared with XPS of Cu_2_Cl(OH)_3_, XPS of Cu_9_S_8_ (red line) revealed increased S 2p and S 2s at 168.33 eV and 232.35 eV, respectively. High-resolution XPS showed that Cu 2p 1/2 and Cu 2p 3/2 were valence states in Cu (Fig. 3G), whereas S2p 1/2 and S2p 3/2 were valence states in S2p (Fig. 3H). These results indicated that S had been successfully vulcanized by Cu. Cu_9_S_8_ had a strong absorption peak at 1100 nm, suggesting that Cu_9_S_8_ exhibits better photothermal properties at 1064 nm, along with robust tissue penetration. Near-infrared II (1000–1400 nm) lasers can penetrate tissue to a depth of 5 mm, which may enable more extensive tumor treatment than conventional lasers with near-infrared I (700–950 nm) penetration depth of 1 mm [39]. We observed a concentration-dependent photothermal pattern of TCuH when the NaHS concentration was held at 3 mM; the temperature increased with increasing TCuH concentration (0, 25, 75 and 100 µg/mL) after illumination (Fig. 3I). In a power gradient experiment, as shown in Fig. 3J, the temperature of a solution comprising TCuH (100 µg/mL) and NaHS (3 mM) increased to the effective treatment temperature (60 °C) within 300 s when the illumination power was 1.0 W/cm^2^, indicating that TCuH could be used for mild PTT in colon cancer. Additionally, Cu_9_S_8_ exhibited good photothermal stability and negligible changes in temperature during four photothermal laser cycles (Fig. 3K). These results showed that TCuH had good near-infrared II photothermal properties after reaction with H_2_S. The fitted cooling curves (Fig. 3L and M) revealed that the photothermal conversion efficiency (η) of Cu_9_S_8_ was 48.3%. Photothermal images of TCuH (100 µg/mL) and NaHS (3 mM) were collected with a thermal imaging camera under extended 1064 nm laser irradiation (0–7 min). As demonstrated in Fig. 3N, the photothermal images gradually became redder, indicating that the temperature was increasing.

### In Vitro cellular uptake and therapeutic efficacy

**Fig. 4:**
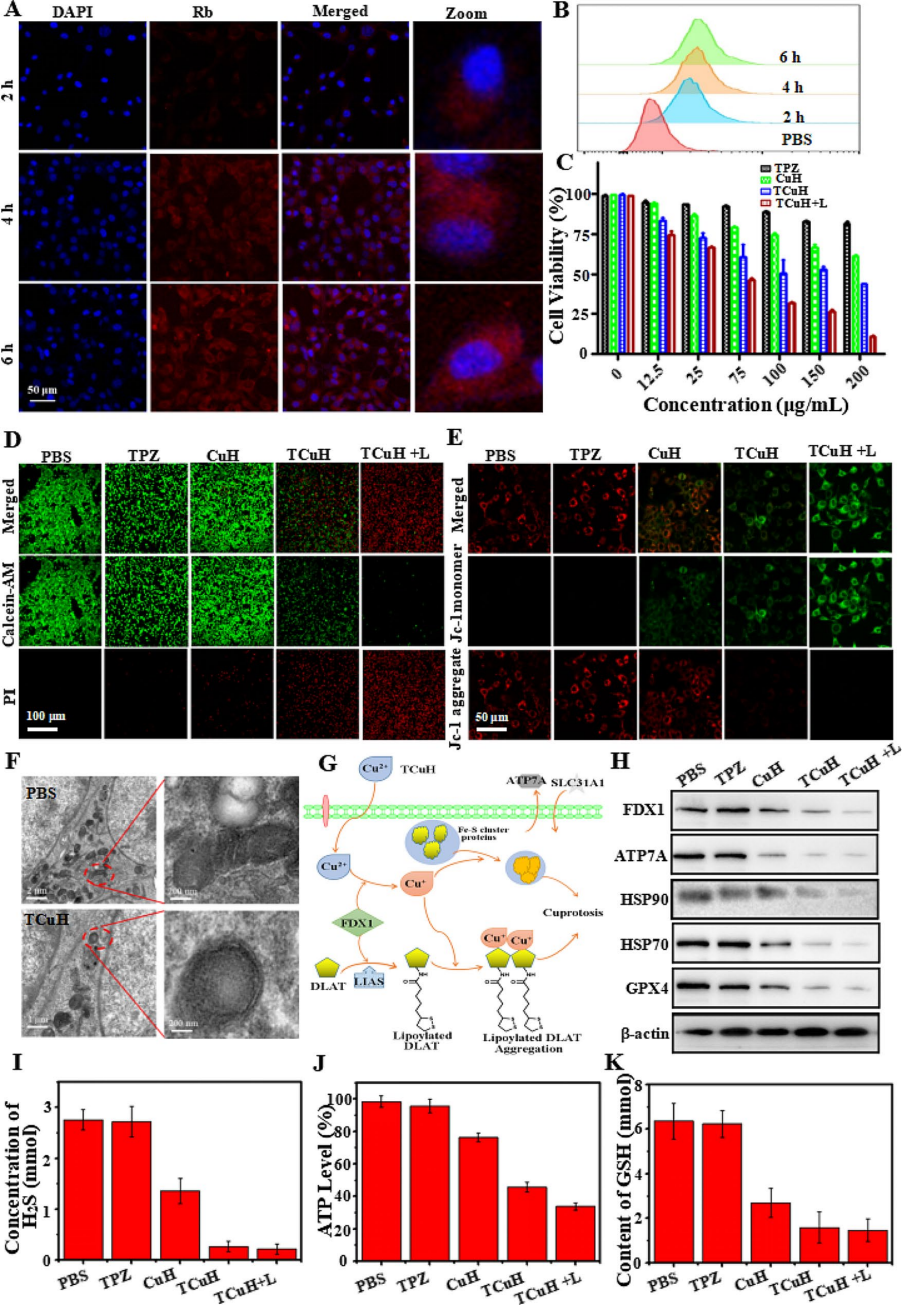
(**A**) CLSM images of CT26 cells treated with RCuH for 2, 4, and 6 h at 37℃, respectively. (**B**) Flow cytometry profiles of CT26 cells incubated with RCuH for 2, 4, and 6 h at 37℃, respectively. (**C**) In vitro MTT-measured toxicities of TPZ, TCu, TCuH, and TCuH + 1064 nm laser (shown as + L in the figure) for 24 h. (**D**) Live/dead assay of CT26 cells incubated with PBS, TPZ, CuH, TCuH and TCuH + L (100 µg/mL) for 6 h. (**E**) Intracellular JC-1 assay of HeLa cells after different treatments with PBS, TPZ, CuH, TCuH and TCuH + L (100 µg/mL) for 6 h. (**F**) Representative Bio-TEM images of CT26 cells before and after treatment with TCuH + L. Red oval indicates location of mitochondria. (**G**) Schematic illustration of the possible mechanism by which TCuH promotes cuproptosis. (**H**) Western blot analysis of expression levels of HSP70, HSP90, FDX1, ATP7A, and GPX4 after incubation with PBS, TPZ, CuH, TCuH, or TCuH + L (100 µg/mL) for 24 h. (**I**) Quantitative comparison of dissolved H_2_S levels among CT26 cells treated with PBS, TPZ, CuH, TCuH, or TCuH + L (100 µg/mL) for 24 h. (**J**) Quantitative comparison of dissolved ATP levels among CT26 cells treated with PBS, TPZ, CuH, TCuH, or TCuH + L (100 µg/mL) for 24 h. (**K**) Quantitative comparison of dissolved GSH levels among CT26 cells treated with PBS, TPZ, CuH, TCuH, or TCuH + L (100 µg/mL) for 24 h

To monitor delivery and intracellular distribution, Rhodamine B (Rb, red fluorescence) was used to assemble RCuH, rather than TPZ. Nuclei were labeled with 4′,6-diamidino-2-phenylindole (DAPI; blue fluorescence) and visualized by a confocal laser scanning microscope (CLSM). As shown in Fig. 4A, an increasing amount of red fluorescence was present in the cytoplasm, suggesting that RCuH was increasingly delivered into cells between 0 and 6 h. The delivery efficiencies (Fig. 4B) of RCuH treatment to CT26 cells were semiquantitatively studied by flow cytometry (FCM). The efficiencies were 23.8%, 48.9%, and 86.5% at 2, 4, and 6 h, respectively. These results confirmed that TCuH could be internalized by cancer cells to achieve therapeutic effects. We immediately measured the viability of TCuH-treated CT26 cells by 3-(4,5-dimethylthiazol-2-yl)-2,5-diphenytetrazolium bromide (MTT) assay. As depicted in Fig. 4C, TPZ did not demonstrate significant cytotoxicity, even at higher concentrations. This result presumably occurred because the tumor site also had a higher O_2_ content, which hindered conversion of TPZ to its active form (TPZ-ed). Regarding CuH, the high expression of GSH at the tumor site can reduce Cu^2+^ to Cu^+^, catalyzing the production of •OH from H_2_O_2_ to induce apoptosis in the tumor site. Therefore, minimal toxicity in CT26 cells was observed at CuH levels of ≤ 200 µg/mL. However, although the endogenous H_2_O_2_ content reached ?∼ 10 mmol, it was insufficient for CDT; thus, the ·OH content was low and CDT had limited cell-killing effects. For similar reasons, TCuH exhibited relatively low toxicity. In contrast, viability was approximately 31.2% when cells were incubated with TCuH (100 µg/mL) and exposed to 1064 nm laser irradiation for 5 min. This result can be explained by the Cu_2_Cl(OH)_3_-mediated consumption of H_2_S to produce photothermic Cu_9_S_8_. When the H_2_S content decreased to 20 µmol, the Scheme 1* reaction occurred in the tumor microenvironment, further reducing the O_2_ content and activating TPZ to generate TPZ-ed. Importantly, the low O_2_ levels hindered ATP production, preventing cells from producing sufficient HSPs to tolerate other stimuli. The decrease in HSPs provided favorable conditions for enhancing the efficacy of mild PTT. Furthermore, PTT can enhance the effects of CDT. The increased amount of Cu^+^ in tumor cells induces the formation of Fe-S clusters, resulting in cuproptosis. Importantly, Cu^2+^ binds to lipoacylated dihydrolipoamide S-acetyltransferase (DLAT), inducing DLAT heteropolymerization. The resulting insoluble DLAT has cytotoxic effects and induces cell death, leading to cuproptosis. Simultaneously, Cu^2+^ generates highly catalytic Cu^+^ under conditions of increased GSH expression, which can catalyze H_2_O_2_ to produce highly toxic hydroxyl radicals (·OH) during chemodynamic therapy (CDT), thereby inducing apoptosis and promoting cuproptosis. To confirm the reactive oxygen species (ROS) production capacity of TCuH NPs in CT26 cells. The non-fluorescent 2’, 7’-dichlorofluorescein diacetate (DCFH-DA) was used as a ROS indicator based on the conversion of 2’, 7’-dichlorofluorescein (DCF, green fluorescence). As shown in Figure SI 4A, we measured the average intensity of ROS signals in CT26 cells treated with various NPs (PBS, TPZ, TCu, TCuH, and TCuH + 1064 nm laser). In the PBS and TPZ groups, CT26 cells stained with DCFH-DA showed very weak or no green fluorescence under a CLSM; conversely, the TCu, TCuH, and TCuH + 1064 nm laser groups exhibited obvious green fluorescence. Therefore, the consumption of endogenous H_2_S promotes •OH formation. Importantly, the TCuH + 1064 nm laser group exhibited stronger fluorescence than the TCu and TCuH groups, suggesting that the photothermal effect of TCuH after the H_2_S reaction promoted •OH production in CT26 cells. Additionally, quantitative analysis of •OH generation in different groups was performed using Image-Pro Plus 6.0. As shown in Figure SI 4B, the average fluorescence intensity in the TCuH + 1064 nm group was 3.58-fold greater than in the TCu group and 2.12-fold greater than in the TCuH group. These results indicated that H_2_S depletion and the photothermal effect could promote •OH formation in CT26 cells. Furthermore, to visually observe live/dead status among CT26 cells in the above groups, live cells were stained with calcein-AM (green fluorescence) and dead cells were stained with propidium iodide (red fluorescence). As shown in Fig. 4D, cells in the PBS, TPZ, and TCu groups showed minimal red fluorescence, indicating that PBS, TPZ, and TCu alone could not effectively kill CT26 cells. However, co-incubation with TCuH and TCuH + 1064 nm laser constituted synergistic treatment, which led to extensive cell death. In the TCuH + 1064 nm laser group, ROS production and the chemotherapeutic effect of TPZ were enhanced; nearly all cells died. FCM analysis indicated that under laser-irradiated TCuH conditions, the apoptosis rate reached 96.5%; this was 8.55-fold, 4.98-fold, and 1.86-fold higher than in the TPZ, TCu, and TCuH groups, respectively (Figure SI 5 A and B). Thus, the combined use of CDT with cuproptosis, PTT, and chemotherapy produced a greater killing effect than any of the treatments alone.

HeLa cells mitochondrial dysfunction was treated with PBS, TPZ, CuH, TCuH and TCuH + L (100 µg/mL) for 6 h by JC-1 assay. As shown in Fig. 4E, the mitochondria membrane of PBS or TPZ-treated Hela cells showed bright red fluorescence, indicating that the mitochondria were in a high-potential, healthy state. Oppositely, TCuH + L significantly depolarized mitochondrial membrane potential showing the highest fluorescence of green, demonstrating mitochondria were dysfunctional, unhealthy status and low potential. In addition, CuH and TCuH treated with Hela cells displayed two kinds of fluorescence (green monomer and red aggregate), suggesting that mitochondrial function wsa sub-optimal health.The morphological changes in CT26 cells treated with PBS or TCuH + 1064 nm laser were further explored by Bio-TEM. Figure 4F shows that mitochondria were intact with in PBS group, whereas severe damage was present among mitochondria in the TCuH + 1064 nm laser group; the damage mainly consisted of increased membrane density, overall mitochondrial shrinkage, and reduced or absent mitochondrial cristae. Cuproptosis contributed to TCuH-induced cell death through the downregulation of Fe-S cluster proteins (Fig. 4G). For example, the reductase FDX1 acts as an upstream regulator of protein thioacylation (e.g., involving DLAT). Additionally, FDX1 reduces Cu^2+^ to Cu^+^, leading to the inhibition of Fe-S cluster protein synthesis and induction of cuproptosis [40]. There are two requirements for cuproptosis: copper content and copper release [41]. Figure 4H shows the expression levels of cuproptosis-related biomarkers (HSP70, HSP90, FDX1, and ATP7A) in CT26 cells treated with PBS, TPZ, TCu, TCuH, or TCuH + 1064 nm laser, as determined by western blotting. The protein expression of HSP70/90 should gradually increase with increasing copper content, thereby enhancing the effect of PTT. However, the in-situ copper ion-based consumption of H_2_S in the tumor led to activation of the Scheme 1 * reaction, promoting O_2_ consumption and decreasing ATP synthesis. A similar trend was observed for ATP7A, which lowered the expression levels of copper-specific efflux proteins. There was sufficient copper loading to promote the occurrence of cuproptosis. Concurrently, Cu^2+^ was reduced to Cu^+^ under conditions of increased GSH expression, which catalyzed •OH production from H_2_O_2_ and diminished GPX4 protein levels. Figure 4I shows the change in H_2_S content after CT26 cells had been incubated with PBS, TPZ, TCu, TCuH, or TCuH + 1064 nm laser. The H_2_S absorbance at 450 nm was measured by an enzyme-labeling method, and the H_2_S concentration was calculated. The H_2_S content in CT26 cells was almost unchanged after TPZ treatment, and it was reduced by 50.9% after TCu treatment. However, the H_2_S contents in CT26 cells treated with TCuH and TCuH + 1064 nm laser were reduced by 90.4% and 92.3%, respectively. These results indicated that after enrichment of a large number of copper carriers in CT26 cells, depletion of a large amount of H_2_S promotes O_2_ depletion, activating the hypoxia prodrug to become active TPZ-ed while inhibiting ATP synthesis (Fig. 4J). Briefly, after sufficient depletion of H_2_S content, mitochondrial reprogramming was induced, which stimulated oxygen consumption by colonic epithelial cells. Moreover, H_2_S oxidation in mitochondria altered cellular bioenergetics, inducing reductive transition that involved NAD/NADH redox pairs. Subsequently, the lack of electron receptors led to deficiencies in uridine and L-aspartic acid, as well as the enhancement of L-glutamine-dependent reductive carboxylation; these conditions exacerbated hypoxia in the tumor microenvironment. The GSH of CT26 cells treated with PBS, TPZ, TCu, TCuH and TCuH + 1064 nm laser. As shown in Fig. 4K, Cu^2+^ reduction to Cu^+^ was diminished under conditions of increased GSH expression, further catalyzing •OH production from H_2_O_2_.

### Antitumor efficacy of TCuH in vivo

**Fig. 5:**
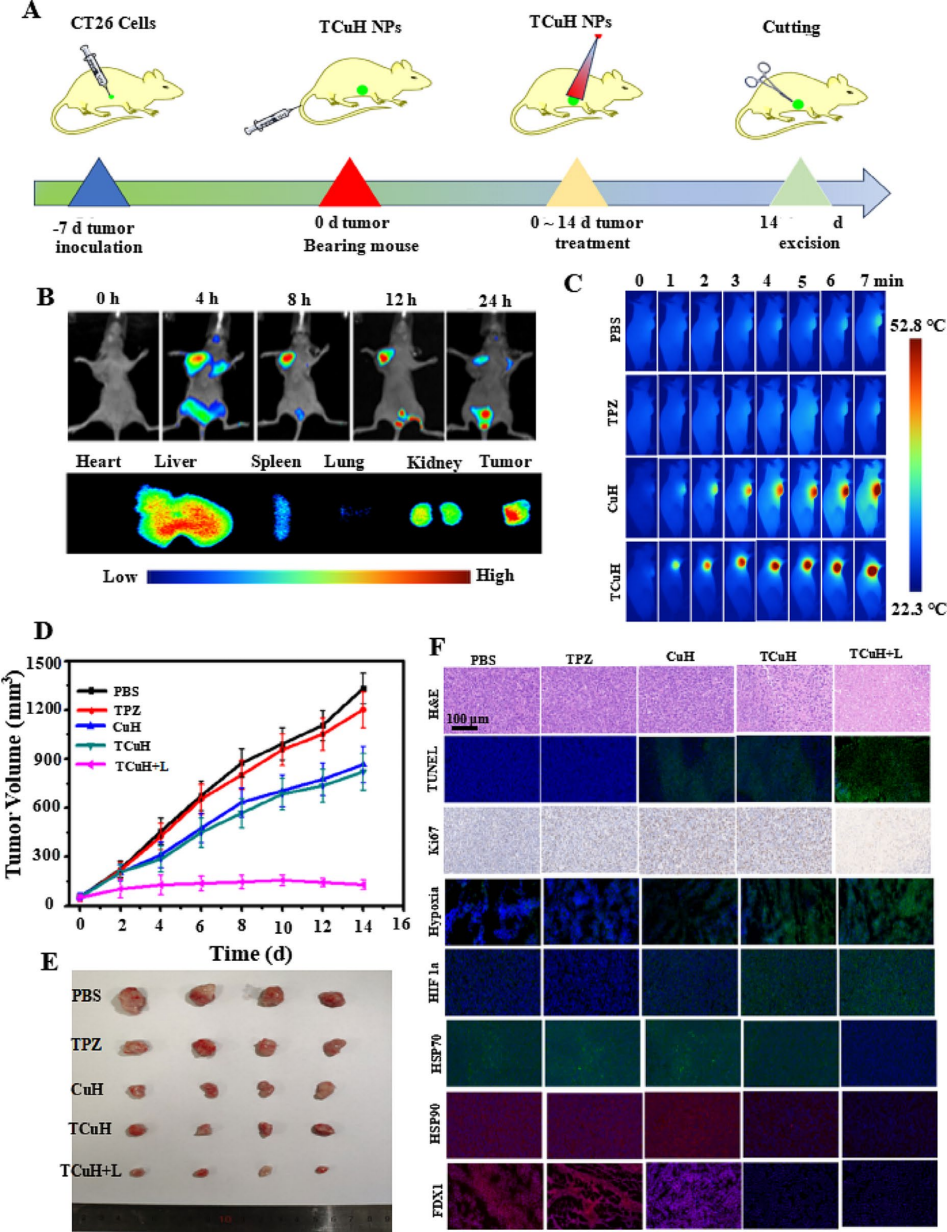
In vivo immune activation and exertion of potent antitumor efficacy. (**A**) Schematic of the treatment protocol. Arrows indicate times of intravenous injection (TCuH NPs, *n* = 5). (**B**) Real-time bio-distribution images of ICuH NPs before and after injection. (**C**) In vivo infrared thermal imaging images of CT26 tumors in mice treated with TCuH NPs upon 1064 nm laser irradiation at 8 h after injection. (**D**) Tumor growth assessment and (**E**) photographs of excised tumor tissues. (**F**) H&E, TUNEL, Ki67, hypoxia, HIF-1a, HSP70, HSP90, and FDX1 staining in tumor sections from PBS, TPZ, TCu, TCuH, and TCuH + 1064 nm laser groups. Data are presented as means with standard deviations (*n* = 5)

We used CT26 cells to establish a subcutaneous tumor model in BALB/c nude mice, with the goal of characterizing the synergistic inhibitory effect of TCuH on colon cancer. All animal procedures were performed in accordance with the Guidelines for Care and Use of Laboratory Animals of National Institutes of Health (NIH publication no. 85 − 23, revised 1996) and approved by the Animal Ethics Committee of Sun Yat-sen University (Guangzhou, China). The mice were randomly divided into five groups (*n* = 5 mice per group): PBS, TPZ, TCu, TCuH, and TCuH + 1064 nm laser. Treatments were administered through the tail vein, once every other day for five total treatments; the total dose was 5 mg/kg (Fig. 5A). Next, in vivo fluorescence imaging techniques were used to track dynamic in vivo changes in TCuH; this allowed determination of the appropriate timing for laser irradiation to induce PTT in the presence of H_2_S. The fluorescence agent indocyanine green (ICG) was used (rather than TPZ) to synthesize ICuH; fluorescence signal aggregation after tail vein injection was observed in the tumor by a small animal imaging instrument. As depicted in Fig. 5B, the ICG fluorescence signal intensity reached a peak at 12 h after injection, then gradually decreased. The fluorescence peak of ICG was present in the tumor at 36 h after dissection, indicating that TCuH can accumulate in tumors for up to 36 h. The liver and kidney have contained large amounts of ICuH that could easily be metabolized by the kidney and reduce toxic side effects. Consequently, we set the laser irradiation time of TCuH to 12 h after injection. As shown in Fig. 5C, the tumor temperature rapidly increased after 1064 nm laser irradiation, indicating a high rate of TCuH activation under a high concentration of H_2_S. In the PBS and TPZ groups, the temperature generally remained below 30℃ with ≤ 7 min of irradiation, indicating that TPZ was not an effective photothermal agent. In mild PTT, the treatment temperature was set to approximately 52.8 °C and the drug was injected into the tail vein for 12 h. The Cu contents in blood and tumor were determined by inductively coupled plasma mass spectrometry (ICP-MS). The results showed that the Cu content in blood gradually decreased to 10 µg/mL after approximately 12 h, with a blood circulation half-life of 2.58 h (Figure SI 6); the Cu content in the tumor gradually decreased after ?∼ 12 h (Figure SI 7). At 12 h after tail vein injection, the tumors were treated with mild PTT involving a single instance of 1064 nm laser irradiation; the temperature reached ?∼ 52.8 °C. Weight and tumor size were measured every other day for 14 days of treatment. Concurrently, TCuH was injected every other day, and 1064 nm laser irradiation was performed 12 h after injection. As shown in Fig. 5D, tumors in the TPZ and PBS groups exhibited nearly identical sizes. In contrast, the rates of inhibition in the TCu, TCuH, and TCuH + 1064 nm laser groups after 14 days of treatment were 35.5%, 39.4%, and 92.3%, respectively. The results indicated that TCuH can produce Cu_9_S_8_ with excellent photothermal conversion at high concentrations of H_2_S, which can inhibit tumor growth in combination with H_2_S-driven chemotherapy and mild photothermal therapy induced mitochondrial reprogramming to promote cuproptosis. There were no significant changes in mouse body weight during the different treatments. In vitro tumor weights and image showed that the results of treatment with different materials were similar to the findings depicted in Fig. 5D, confirming the excellent antitumor effect of TCuH + 1064 nm laser treatment. Mouse body weight steadily increased, indicating no significant delay in tumor growth (Figure SI 8). Notably, the TCuH group displayed the greatest inhibitory effect on tumor growth, with a statistically significant difference relative to the other groups (Figures SI 9 and Fig. 5E).

To explain the potential therapeutic mechanism of TCuH, histological analysis was performed to investigate TCuH molecular functions in vivo. Compared with the PBS group, the TPZ, TCu, TCuH, and TCuH + 1064 nm laser groups demonstrated higher terminal deoxynucleotidyl transferase-mediated dUTP nick-end labeling (TUNEL) and hematoxylin and eosin (H&E) signals of apoptosis, along with a lower percentage of Ki67-positive cells (Fig. 5F). Apoptosis and necrosis among tumor cells were greater in the TCuH + 1064 nm laser group under H_2_S activation, compared with the other groups. To explore the antitumor mechanism of TCuH + 1064 nm laser under H_2_S activation, in vivo immunofluorescence assays were used to measure hypoxia and the expression levels of HIF-1a, HSP70, HSP90, and FDX1 in tumors. Consistent with the in vitro results, the most prominent aggregation of HIF-1a and highest level of hypoxia were observed in tumors treated with TCuH + 1064 nm laser under H_2_S activation. Higher green fluorescence intensity indicated lower O_2_. As The high-affinity Cu ions reacted with excess H_2_S in the tumor, resulting in a rapid reduction in H_2_S content to < 20 µmol, which activated mitochondrial reprogramming and stimulated oxygen consumption by colonic epithelial cells. Subsequently, the lack of electron receptors led to deficiencies in uridine and L-aspartic acid, as well as the enhancement of L-glutamine-dependent reductive carboxylation; these conditions exacerbated hypoxia in the tumor microenvironment (Scheme 1*). Heat shock protein (HSP70/90) is an ATP-dependent chaperone, and ATP synthesis is inhibited when O_2_ levels are reduced. Therefore, the HSP70/90 content decreases with decreasing O_2_ content. This is also a prerequisite for mild PTT therapy. Moreover, loss of FDX1 was observed in tumors treated with TCuH + 1064 nm laser under H_2_S activation, confirming the presence of cuproptosis in vivo. The results showed that TCuH + 1064 nm laser treatment could effectively inhibit tumor growth via H_2_S-driven chemotherapy and mild photothermal therapy induced mitochondrial reprogramming to promote cuproptosis.

### Biosafety evaluation of TCuH NPs

**Fig. 6:**
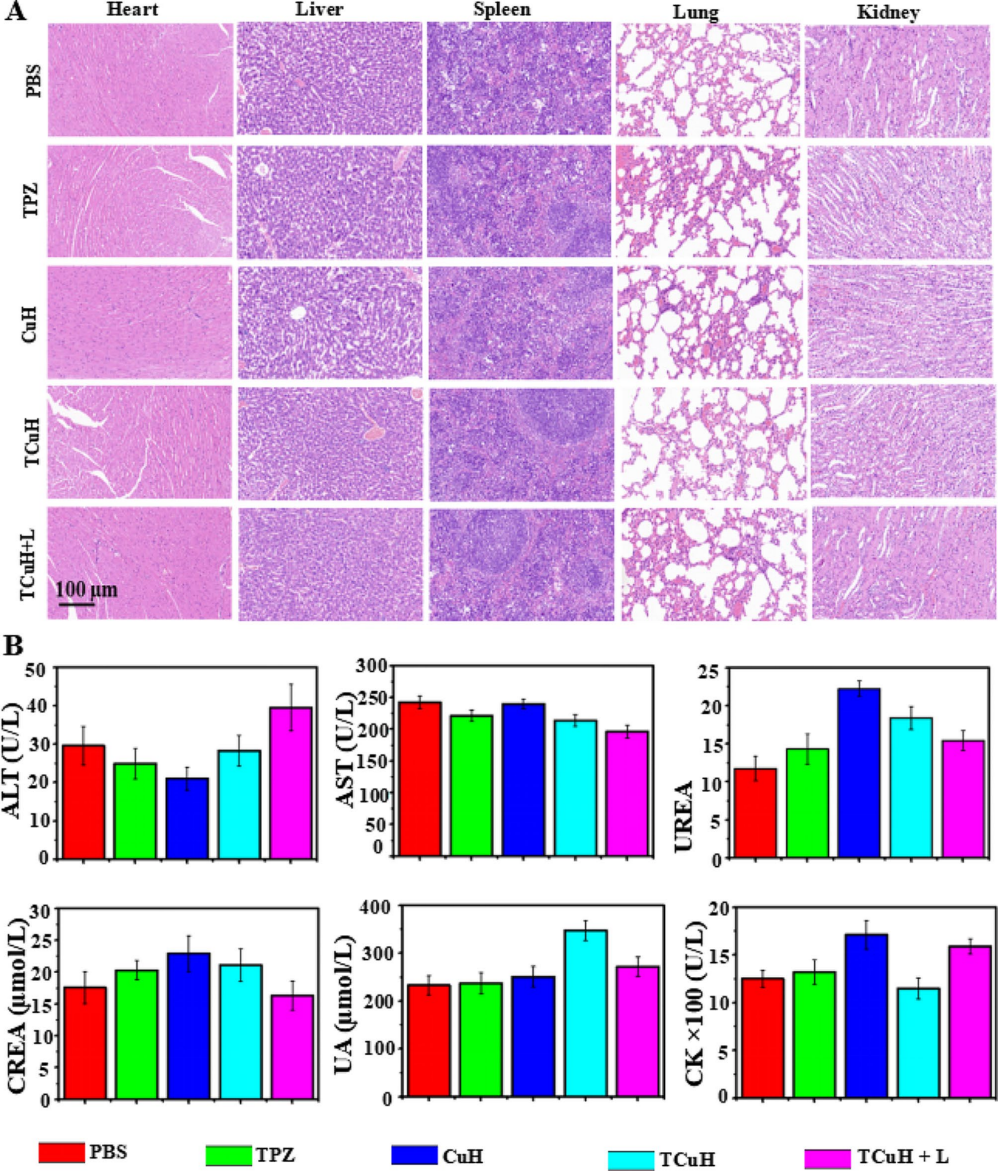
(**A**) H&E staining images of major organs isolated from mice after treatment with PBS, TPZ, CuH, TCuH, or TCuH + L. (**B**) In vivo biological safety evaluated by biochemical analyses of blood and serum after treatment (*n* = 3 per group) with PBS, TPZ, CuH, TCuH, or TCuH + L

Although the cytotoxicity of TCuH + 1064 nm treatment was difficult to detect through histological assays, its practical application requires consideration of potential side effects. Consequently, PBS, TPZ, CuH, TCuH and TCuH + 1064 nm laser treatments were injected into healthy mice through the tail vein (total dose, 5 mg/kg) to explore biosafety in vivo. As depicted in Fig. 6A, H&E staining of major organs showed no morphological changes in any group, suggesting no obvious side effects of TCuH NPs and sufficient safety in vivo. We also investigated the blood physiological and biochemical parameters of mice in each treatment group. The results (Fig. 6B) showed that alanine transaminase, urea, serum creatinine, blood uric acid, and creatine kinase did not significantly differ among groups, confirming that the CuH NPs have minimal toxicity.

## Conclusion

In summary, we constructed H_2_S-driven TCuH NPs in a multifunctional cascade bioreactor with enhanced efficacy for targeted cancer therapy by inducing mitochondrial reprogramming to promote cuproptosis. After colon cancer had been treated with TCuH NPs, H_2_S content was depleted, resulting in mesoporous Cu_2_Cl(OH)_3_ cleavage and release of the hypoxic prodrug TPZ, as well as generation of the near-infrared II photothermal agent Cu_9_S_8_. The depletion of H_2_S induced mitochondrial reprogramming and stimulated oxygen consumption in colonic epithelial cells; H_2_S oxidation in mitochondria altered cellular bioenergetics, inducing reductive transition of NAD/NADH redox pairs. Subsequent electron receptor deficiency led to deficiencies in uridine and L-aspartic acid and the enhancement of L-glutamine-dependent reductive carboxylation, exacerbating hypoxia in the tumor microenvironment. Activation of the hypoxic prodrug TPZ produced activated TPZ-ed, which had chemotherapeutic effects on colon cancer. Additionally, hypoxia inhibited ATP synthesis, resulting in decreased expression of HSPs (HSP70 and HSP90), and improving the effects of temperature treatment and PTT. Colon cancer is also enriched in copper ions and displayed cuproptosis, mediated by FDX1. Furthermore, Cu^2+^ was reduced to Cu^+^ with high catalytic activity under conditions of increased GSH expression, which facilitate ·OH production from H_2_O_2_ and the onset of apoptosis. Overall, we successfully prepared H_2_S-driven TCuH NPs to promote chemotherapy/CDT/mild PTT/cuproptosis therapy for colon cancer by inducing mitochondrial reprogramming. An important limitation was that we did not validate the findings in a mouse model of in situ colon cancer.

### Electronic supplementary material

Below is the link to the electronic supplementary material.

Supplementary Material 1

Supplementary Material 2

## Data Availability

No datasets were generated or analysed during the current study.

## References

[1] Untereiner AA, Pavlidou A, Druzhyna N, Papapetropoulos A, Hellmich MR, Szabo C (2018). Drug resistance induces the upregulation of H_2_S-producing enzymes in HCT116 colon cancer cells. Biochem Pharmacol.

[2] Marmol I, Sanchez-de-Diego C, Pradilla Dieste A, Cerrada E, Rodriguez Yoldi MJ (2017). Colorectal carcinoma: a general overview and future pperspectives in colorectal ccancer. Int J Mol Sci.

[3] Israelsen M, Madsen BS, Torp N, Johansen S, Hansen CD, Detlefsen S, Andersen P, Hansen JK, Lindvig KP, Rasmussen DN (2023). Rifaximin-alpha for liver fibrosis in patients with alcohol-related liver disease (GALA-RIF): a randomised, double-blind, placebo-controlled, phase 2 trial. Lancet Gastroenterol Hepatol.

[4] Sunakawa Y, Bekaii-Saab T, Stintzing S (2016). Reconsidering the benefit of intermittent versus continuous treatment in the maintenance treatment setting of metastatic colorectal cancer. Cancer Treat Rev.

[5] Jiang X, Lee M, Xia J, Luo T, Liu J, Rodriguez M, Lin W (2022). Two-stage SN38 release from a core-shell nanoparticle enhances tumor deposition and antitumor efficacy for synergistic combination with immune checkpoint blockade. ACS Nano.

[6] Ma S, Song W, Xu Y, Si X, Zhang D, Lv S, Yang C, Ma L, Tang Z, Chen X (2020). Neutralizing tumor-promoting inflammation with polypeptide- dexamethasone conjugate for microenvironment modulation and colorectal cancer therapy. Biomaterials.

[7] Szabo C, Coletta C, Chao C, Modis K, Szczesny B, Papapetropoulos A, Hellmich MR (2013). Tumor-derived hydrogen sulfide, produced by cystathionine-beta-synthase, stimulates bioenergetics, cell proliferation, and angiogenesis in colon cancer. Proc Natl Acad Sci.

[8] Chen W, Ni D, Rosenkrans ZT, Cao T, Cai W (2019). Smart H_2_S-triggered/therapeutic system (SHTS)-based nanomedicine. Adv Sci.

[9] An L, Wang X, Rui X, Lin J, Yang H, Tian Q, Tao C, Yang S (2018). The in situ sulfidation of Cu_2_O by endogenous H_2_S for ccolon cancer theranostics. Angew Chem Int Ed Engl.

[10] Zatarain JR, Mrazek AA, Johnson P, Pang L, Ding Y, Zhou J, Szabo C, Chao C, Hellmich MR (2015). Tu1975 H_2_S inhibition of cystathionine-β-synthase (CBS) using novel prodrug decreases colorectal cancer xenograft growth with less toxicity than aminooxyacetic acid (AOAA). Gastroenterology.

[11] Ascencao K, Szabo C (2022). Emerging roles of cystathionine beta-synthase in various forms of cancer. Redox Biol.

[12] Tilg H, Adolph TE, Gerner RR, Moschen AR (2018). The intestinal microbiota in colorectal cancer. Cancer Cell.

[13] Zhu K, Qian S, Guo H, Wang Q, Chu X, Wang X, Lu S, Peng Y, Guo Y, Zhu Z (2022). pH-activatable organic nanoparticles for efficient low-temperature photothermal therapy of ocular bacterial infection. ACS Nano.

[14] Yang L, Zhu Y, Liang L, Wang C, Ning X, Feng X (2022). Self-assembly of intelligent nanoplatform for endogenous H_2_S-Triggered multimodal cascade therapy of ccolon cancer. Nano Lett.

[15] Zhou R, Xu H, Qu J, Ohulchanskyy TY (2023). Hemoglobin nanocrystals for drugs free, synergistic theranostics of colon tumor. Small.

[16] Li Y, Chen W, Qi Y, Wang S, Li L, Li W, Xie T, Zhu H, Tang Z, Zhou M (2020). H_2_S-scavenged and activated iron oxide-hydroxide nanospindles for MRI-guided photothermal therapy and ferroptosis in colon cancer. Small.

[17] Liu C, Han Y, Zhang J, Kankala R, Wang S, Chen A (2019). Rerouting engineered metal-dependent shapes of mesoporous silica nanocontainers to biodegradable Janus-type (sphero-ellipsoid) nanoreactors for chemodynamic therapy. Chem Eng J.

[18] Qiao L, Li X, Xiao Y, Yuan J, Yu D, Zuo M, Chen J, Han S, Cheng D (2022). A component-optimized chemo-dynamic nanoagent for enhanced tumour cell-selective chemo-dynamic therapy with minimal side effects in a glioma mouse model. Biomater Sci.

[19] Zhao Y, Chen B, Kankala R, Wang S, Chen A (2020). Recent advances in combination of copper chalcogenide-based photothermal and reactive oxygen species-related therapies. ACS Biomat Sci Eng.

[20] Zou B, Xiong Z, He L, Chen T (2022). Reversing breast cancer bone metastasis by metal organic framework-capped nanotherapeutics via suppressing osteoclastogenesis. Biomaterials.

[21] Meng R, Zhao Y, Xia H, Wang S, Chen A, Kankala R (2024). 2D architectures- transformed conformational nanoarchitectonics for light-augmented nanocatalytic chemodynamic and Photothermal/Photodynamic-Based Trimodal therapies. ACS Mater Lett.

[22] Cheng J, Zhu Y, Dai Y, Li L, Zhang M, Jin D, Liu M, Yu J, Yu W, Su D (2023). Gas-mediated tumor energy remodeling for sensitizing mild photothermal therapy. Angew Chem Int Ed Engl.

[23] Wang C, Xue F, Wang M, An L, Wu D, Tian Q (2022). 2D Cu-Bipyridine MOF Nanosheet as an Agent for Colon cancer therapy: a three-in-one Approach for Enhancing Chemodynamic Therapy. ACS Appl Mater Inter.

[24] Niu X, Zhu Y, Ding C, Ma J, Wei P, Lin Y, Fang W, He Q, Li C, Cheng J et al. Dual-active Center AgFeCu Nanocatalyst for Tumor Destruction via Self‐catalytically enhanced mild Photothermal Therapy. Adv Funct Mater 2023, 2306778.

[25] Libiad M, Vitvitsky V, Bostelaar T, Bak DW, Lee HJ, Sakamoto N, Fearon E, Lyssiotis CA, Weerapana E, Banerjee R (2019). Hydrogen sulfide perturbs mitochondrial bioenergetics and triggers metabolic reprogramming in colon cells. J Biol Chem.

[26] Hildebrandt TM, Grieshaber MK (2008). Three enzymatic activities catalyze the oxidation of sulfide to thiosulfate in mammalian and invertebrate mitochondria. FEBS J.

[27] Libiad M, Yadav PK, Vitvitsky V, Martinov M, Banerjee R (2014). Organization of the human mitochondrial hydrogen sulfide oxidation pathway. J Biol Chem.

[28] Grzelak A, Wojewódzka M, Meczynska-Wielgosz S, Zuberek M, Wojciechowska D, Kruszewski M (2018). Crucial role of chelatable iron in silver nanoparticles induced DNA damage and cytotoxicity. Redox Biol.

[29] Guo B, Yang F, Zhang L, Zhao Q, Wang W, Yin L, Chen D, Wang M, Han S, Xiao H, Xing N (2023). Cuproptosis induced by ROS responsive nnanoparticles with elesclomol and copper combined with alphaPD-L1 for enhanced cancer immunotherapy. Adv Mater.

[30] Hu H, Xu Q, Mo Z (2022). New anti-cancer explorations based on metal ions. J Nanobiotechnol.

[31] Sun L, Zhang Y, Yang B, Sun S, Zhang P, Luo Z, Feng T, Cui Z, Zhu T, Li Y (2023). Lactylation of METTL16 promotes cuproptosis via m(6)A-modification on FDX1 mRNA in gastric cancer. Nat Commun.

[32] Chen W, Xie W, Gao Z, Lin C, Tan M, Zhang Y, Hou Z (2023). Mild-photothermal effect induced high efficiency ferroptosis-boosted-cuproptosis based on Cu_2_O@Mn_3_Cu_3_O_8_ Nanozyme. Adv Sci.

[33] Xu Y, Liu SY, Zeng L, Ma H, Zhang Y, Yang H, Liu Y, Fang S, Zhao J, Xu Y (2022). An enzyme-engineered nonporous copper(I) coordination polymer nanoplatform for cuproptosis-based synergistic cancer therapy. Adv Mater.

[34] Di X, Pei Z, Pei Y, James TD (2023). Tumor microenvironment-oriented MOFs for chemodynamic therapy. Coordin Chem Rev.

[35] He Y, Hua Liu S, Yin J, Yoon J (2021). Sonodynamic and chemodynamic therapy based on organic/organometallic sensitizers. Coordin Chem Rev.

[36] Cheng Y, Bo H, Qin R (2022). Hyaluronic acid-coated Bi:Cu_2_O: an H_2_S-responsive agent for colon cancer with targeted delivery and enhanced photothermal performance. J Nanobiotechnol.

[37] Guo G, Zhang H, Shen H, Zhu C, He R, Tang J, Wang Y, Jiang X, Wang J, Bu W, Zhang X (2020). Space-selective chemodynamic therapy of CuFe_5_O_8_ nanocubes for implant-related infections. ACS Nano.

[38] Wang M, Chang M, Chen Q, Wang D, Li C, Hou Z, Lin J, Jin D, Xing B (2020). Au(2)Pt-PEG-Ce6 nanoformulation with dual nanozyme activities for synergistic chemodynamic therapy/phototherapy. Biomaterials.

[39] Lu H, Carroll GM, Neale NR, Beard MC (2019). Infrared Quantum dots: Progress, challenges, and opportunities. ACS Nano.

[40] Tsvetkov P, Coy S, Petrova B, Dreishpoon M, Verma A, Abdusamad M, Rossen J, Joesch-Cohen L, Humeidi R, Spangler R, Eaton J, Frenkel E, Kocak M, Corsello S, Lutsenko S, Kanarek N, Santagata S (2022). Copper induces cell death by targeting lipoylated TCA cycle proteins. Science.

[41] Xu W, Qian J, Hou G, Wang T, Wang J, Wang Y, Yang L, Cui X, Suo A (2022). A hollow amorphous bimetal organic framework for synergistic cuproptosis/ferroptosis/apoptosis anticancer therapy via disrupting intracellular redox homeostasis and copper/iron metabolisms. Adv Funct Mate.

