# Supplementary material for: H2S-driven chemotherapy and mild photothermal therapy induced mitochondrial reprogramming to promote cuproptosis
Source: J Nanobiotechnology. 2024 Apr 24;22:205. doi: 10.1186/s12951-024-02480-x (PMC11044430; doi:10.1186/s12951-024-02480-x)
Supplement: Supplementary file 1 — Supplementary Material 1 [file 12951_2024_2480_MOESM1_ESM.docx]

**Supporting Information**

**H_2_S-driven Chemotherapy and Mild Photothermal Therapy Induced Mitochondrial Reprogramming to Promote Cuproptosis**

Lihong Qiao ^1, 2, 3, 4^, ^#^ Yijing Ou ^1, 2^, ^#^ Lin Li ^1, 2^, ^#^ Shuzhen Wu ^1, 2^, Yanxian Guo ^1, 2^, Mu Liu ^1, 2^, Dongsheng Yu ^4^, Qinghua Chen ^4^, Jianmin Yuan ^4^, Chuanqi Wei ^4^, Chiyi Ou ^4^, Haowen Li ^3^, Du Cheng ^4^*, Zhiqiang Yu ^3^* and Zhongjun Li ^1, 2^*

^1^ Dongguan Key Laboratory of Major Diseases in Obstetrics and Gynecology，The Tenth Affiliated Hospital of Southern Medical University (Dongguan people's hospital)，Dongguan, Guangdong 523058, People’s Republic of China.

^2^ Department of Obstetrics and Gynecology, The Tenth Affiliated Hospital of Southern Medical University (Dongguan people's hospital), Dongguan 523059, People's Republic of China

^3^ Department of Laboratory Medicine Dongguan Institute of Clinical Cancer Research, The Tenth Affiliated Hospital of Southern Medical University (Dongguan People's Hospital) Dongguan, Guangdong 523058, People's Republic of China

^4^ Key Laboratory for Polymeric Composite & Functional Materials of Ministry of Education, School of Materials Science and Engineering, Sun Yat-sen University, Guangzhou 510275, People's Republic of China.

^#^ These authors contributed equally to this work.

**E-mail:** chengdu@mail.sysu.edu.cn; yuzq@smu.edu.cn and Zhongjun@gdmu.edu.cn.

**Experimental Section**

**Materials:** Cupric chloride (CuCl_2_·2H_2_O), hydrogen peroxide (30% H_2_O_2_), NaOH, tirapazamine (TPZ), hyaluronic acid (HA), rhodamine B, indocyanine green (ICG), 2′,7′-dichlorofluorescin diacetate (DCFH-DA), 3-(4,5-dimethyl-2-thiazolyl)-2,5-diphenyl-2-H-tetrazolium bromide (MTT), sodium hydrosulfide, and DAPI were purchased from Aladdin (China). All antibodies were purchased from Abcam. All reagents were purchased from commercial sources and used without further treatment.

**Characterization:** Fourier transform infrared spectra were recorded on a Bruker Vertex 70 spectrometer. UV-Vis absorption spectra were acquired using a Shimadzu UV-2450 spectrophotometer. An X-ray diffractometer (Bruker AXS D8 Focus) was operated using Cu Ka radiation (λ = 1.54056 Å). Nanoparticle zeta potential was analyzed using a Zetasizer Nano-ZS (Malvern Instruments Ltd.). Transmission electron microscopy (TEM) images were recorded with a FEI-TECNAI G2 transmission electron microscope operating at 120 kV. Electron spin resonance (ESR) spectra were conducted on an EMXplus spectrometer (Bruker, Germany). Inductively coupled plasma-mass spectrometry (ICP-MS) were carried out on an Agilent 7700x series ICP-MS instrument.


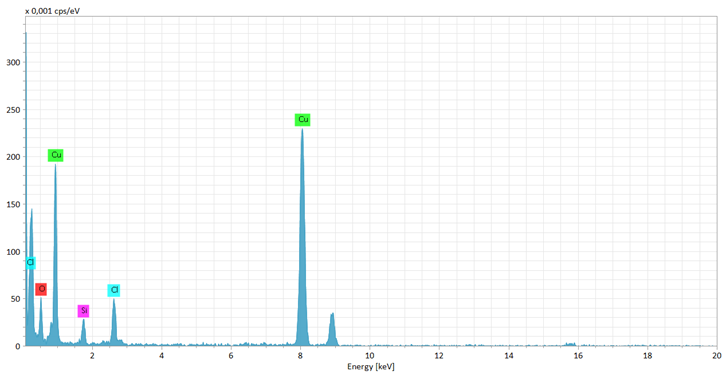


SI 1. EDS of TPZ@CuO NPs.

SI 2. TPZ loading content was recorded by UV-Vis.

SI 3. Cu 2p XPS of TCuH NPs.


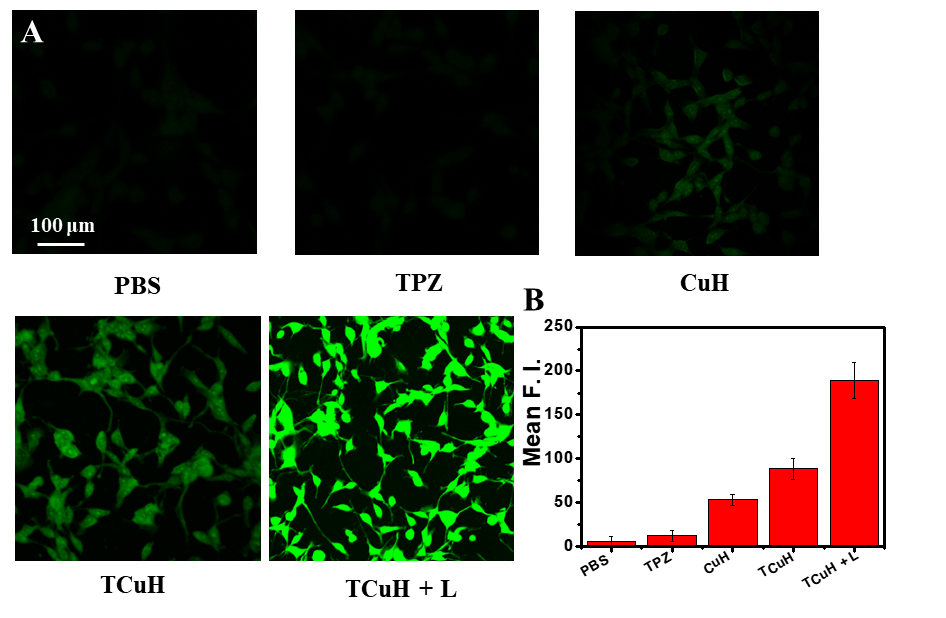


SI 4. (A) CLSM images of CT26 cells after co-incubation with PBS, TPZ, TCu, TCuH, and TCuH + 1064 nm laser for 6 h, followed by staining with the •OH fluorescence probe DCFH-DA. (B) Mean fluorescence intensity (Mean F. I.) image of •OH fluorescence probe.


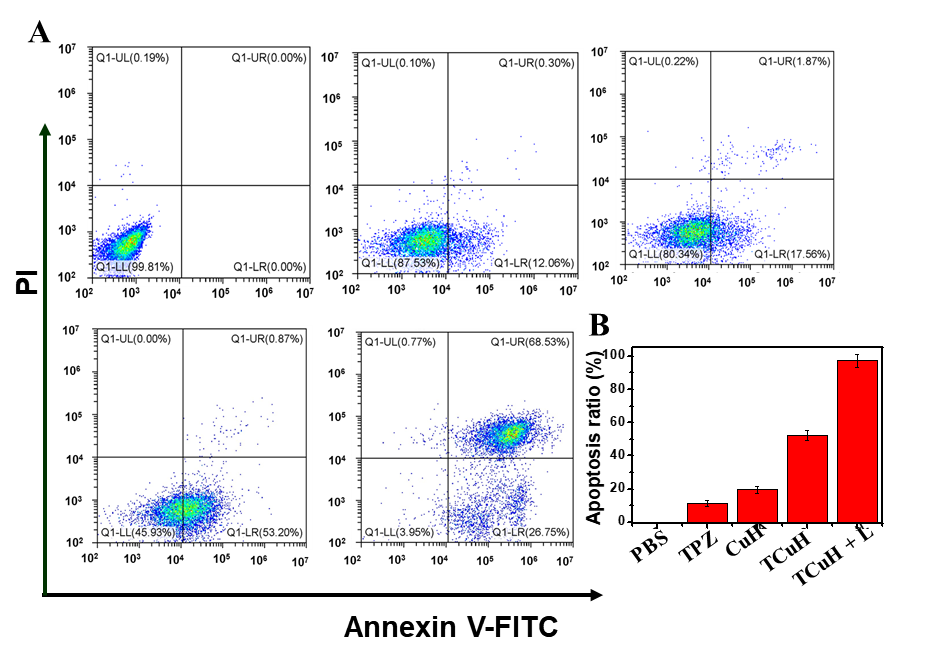


SI 5. (A) Flow cytometry analysis of apoptosis among cells co-incubated with PBS, TPZ, TCu, TCuH, and TCuH + 1064 nm laser (100 µg/mL) for 12 h. (B) Semi-quantification of apoptosis rate among CT26 cells treated with PBS, TPZ, TCu, TCuH, and TCuH + 1064 nm laser(100 µg/mL) via flow cytometry (FCM).


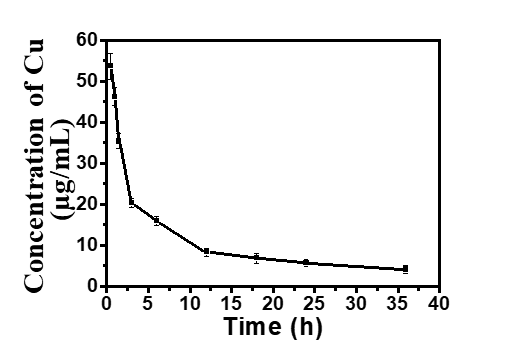


SI 6. Cu content in blood at various time points according to ICP-MS.


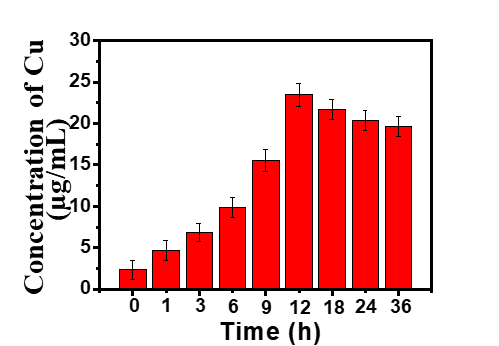


SI 7. Cu content in tumors at various time points according to ICP-MS.

SI 8. Weight (g) curves of mice were treated with PBS, TPZ, TCu, TCuH, and TCuH + 1064 nm laser groups.

SI 9. In vitro tumor weight (g) comparison among all groups.
